# In the Living Room and Across the Screen: Intergenerational Play Between Infants and Grandparents

**DOI:** 10.1111/infa.70065

**Published:** 2026-01-11

**Authors:** Lucinda I. Neely, Douglas J. Piper, Lauren J. Myers, Jennifer M. Zosh, Gabrielle A. Strouse, Georgene L. Troseth, Rachel Barr

**Affiliations:** ^1^ Georgetown University Washington DC USA; ^2^ Lafayette College Easton Pennsylvania USA; ^3^ Penn State Brandywine Media Pennsylvania USA; ^4^ University of South Dakota Vermillion South Dakota USA; ^5^ Vanderbilt University Nashville Tennessee USA

## Abstract

In‐person co‐play between infants and adults develops rapidly during infancy, but little research has examined how families play together over video chat. Research demonstrates that video chat may support family connections, especially with grandparents and other family members separated by physical location. However, video chat interactions also place significant socio‐cognitive demands on infants that may impact the frequency and variety of family play. The present study examines predictors of intergenerational virtual play compared to in‐person play. We conducted an OSF pre‐registered secondary analysis of data from a longitudinal study of 47 infant‐parent‐grandparent triads who recorded up to three naturalistic Zoom video chats and a session when they met in person. All instances of attempted infant‐grandparent play were coded for playful activity type (e.g., dancing, hide & seek), duration, and whether the infant was successfully engaged in play (e.g., responded by smiling, vocalizing or imitating). Descriptive analyses revealed variability in play between families and across sessions. To capture the variety of ways in which grandparents, parents and infants played together, we fit growth models to predict the frequency of play bouts, the number of different types of playful activities observed (play repertoire), the proportion of time engaged in play, the proportion of play bouts for which infants were positively engaged, and the proportion of play bouts initiated by infants during video chat sessions. Across analyses, age was the strongest predictor of infant play on video chat. We then compared video chat play to play during the in‐person session and found that play repertoire was significantly greater on video chat than in person. This study highlights the potential of digital tools to enhance intergenerational family relationships and social interactions through play. Video chat may serve as a high‐quality supplemental activity for separated families.

## 1 Introduction

Children and infants develop optimally in the context of stable and nurturing relationships with parents and caregivers (National Research Council and Institute of Medicine 2000). Infants learn and build close social connections through back‐and‐forth communication with their caregivers (Chen et al. 2023), frequently through serve‐and‐return exchanges during play (Konishi et al. 2014). Play interactions are crucial to infant social skill development as well as cognitive and language skills, creativity, and fine and gross motor development (Ginsburg K. R. et al. 2007; Herzberg et al. 2022).

Grandparent involvement in a child's life has been linked with positive outcomes for both child (Barnett et al. 2010) and family (Poblete and Gee 2018). Although most grandchild‐grandparent studies indicate there are benefits for both children and their grandparents, this research is primarily focused on older grandchildren (e.g., Davey et al. 2009; Duflos et al. 2020; Dunifon 2013; Dunifon and Bajracharya 2012). For example, grandparent involvement in the lives of adolescent grandchildren was associated with greater child well‐being (Griggs et al. 2010), and children's closeness to a grandparent in middle childhood was associated with fewer adjustment problems (Lussier et al. 2002). Here, we extend the focus to very young (infant and toddler) grandchildren and their grandparents and specifically explore how virtual intergenerational connection, in the form of playful interactions on video chat, may provide an additional positive context for development.

For many families, geographic separation makes physical interactions between infants and their grandparents a rare occasion, but families have bridged the distance by using video chat to keep in touch (David and Kakulla 2019). During the COVID‐19 pandemic, 80% of grandparents reported video chatting with their grandchildren before they were 4 months of age, and 82% of grandparents reported that they enjoyed video chatting with their grandchildren (AARP 2021). The American Academy of Pediatrics (2016) has also made video chat an exception to their screen time guidance, emphasizing the value of video chat for supporting family connections. Although research demonstrates that video chat frequency may be associated with adults' self‐reported feelings of connectedness with their grandchildren (Strouse et al. 2021), little is known about the specific activities they share over video chat to support that closeness.

Given the central role of play for infant socio‐emotional development and the increasing frequency of virtual interactions in the lives of young children, the present study investigated intergenerational playful activities that occurred across the screen while families video chatted. Play on video chat requires consideration of several complexities introduced when the play partner is not physically present but appears on a video screen. For example, imagine Grandma on the screen playing peek‐a‐boo with the baby who is watching the screen of a mobile phone or computer. The on‐screen person can respond to the infant in real‐time, but she is not there in the room. Here, we examined how intergenerational play on video chat compared to play during an in‐person meeting between the same child and grandparent.

### 1.1 Infant Play

Play emerges and develops rapidly during the infancy period (see Piaget 1962 for seminal work). During in‐person interactions, play occurs along a spectrum from free play (unstructured play where children have full autonomy) to guided play (where adults scaffold learning within a playful context; Zosh et al. 2018). Play during infancy also varies in context and content, including sensorimotor and object play, physical or locomotor play, rough‐and‐tumble play, exploratory play, construction play, and symbolic play (Lillard et al. 2013). For example, sensorimotor play allows infants to explore first their bodies and later, the objects around them, providing opportunities for even the youngest infants to discover their capabilities and learn about objects and their affordances. Object play, particularly frequent and varied interactions with objects, enhances infants' gross and fine motor skills while also supporting perceptual development (Lockman and Tamis‐LeMonda 2021; Rachwani et al. 2020).

In both lab‐based (Kannass et al. 2006; Adolph and Robinson 2015) and semi‐naturalistic observational studies (Herzberg et al. 2022), infant object play is characterized by brief, highly distractible bouts and rapid shifting between objects, with infants exploring dozens of objects per hour in short bursts. Social play, defined as sharing attention with a partner on the same object (Shin 2012), is also a hallmark of play in infancy. During social play, communication between the infant and partner typically occurs through gaze following and eye contact and includes joint attention, an interaction that occurs when two people coordinate their attention to an object (Brooks and Meltzoff 2014; Nomikou et al. 2013). This is a rich context for infants to learn about the world: mothers' use of language and verbalizations to maintain infant attention toward toys at 9 months predicted infants' linguistic skills at 12 and 15 months (Carpenter et al. 1998). In contrast to sensorimotor and social play, pretend play emerges later and requires higher levels of processing and cognitive capabilities as infants engage in symbolic thinking (Lillard et al. 2013). Engagement in a variety of types along the play spectrum provides important ways for infants to learn about and interact with the world around them (Whitebread et al. 2012).

### 1.2 Play on Video Chat

In some ways, play over video chat can be similar to play in face‐to‐face contexts. During video chat, the participating parties can see each other's facial expression, gaze direction, and gestures, which allow them to convey emotion and connect with one another. Social contingency (i.e., people responding to one another in the moment) typically occurs in playful video chat interactions (McClure et al. 2018; Myers et al. 2017; Roseberry et al. 2014), as it does for in‐person shared play. For example, McClure and colleagues (2020) found that young infants (6‐ to 12‐months) who played peek‐a‐boo with their mothers smiled and gazed at their mothers (i.e., responded) as frequently in video chat and in‐person contexts. Infants as young as 8 months have been observed to initiate interactions with people on screen, and initiations increase with age (McClure et al. 2018; Myers et al. 2024). During the second year of life, toddlers were more likely to play and imitate actions with responsive, across‐screen partners who interacted with them via video chat than they were when watching the same people on pre‐recorded videos instructing them to engage in the same activities (Myers et al. 2017; Strouse et al. 2018), showing that young children can be active participants in virtual interactions.

However, video chat also presents both social challenges (e.g., engaging with a partner through a screen) and cognitive challenges (e.g., understanding and representing on‐screen objects; McClure and Barr 2017; Troseth et al. 2024) that may impact how infants and their caregivers play, leading to important differences between in‐person and video chat play. During in‐person object play, infants can physically explore objects from multiple angles as adults follow this exploration and label and discuss the objects, which helps infants learn (Yu and Smith 2013). In comparison, shared object play on video chat presents unique challenges. For example, in the middle of a responsive interaction with their grandmother on video chat, an infant may attempt to pass a toy to her, not realizing that the toy cannot physically go through the screen. *Media errors* such as these are common when children are 8–10 months old (Rosengren et al. 2021). Infants between 9 and 15 months also pat, hit, and try to grasp still and moving objects on the screen (Chiong and Shuler 2010; Pierroutsakos and Troseth 2003). Of course, these images are 2‐dimensional depictions of objects that the infant cannot access and play with in the same way they would play with 3‐dimensional toys. During video chat, the toy that Grandma shows and discusses on the screen has to compete for attention with objects in the infant's environment.

Shared play on video chat also may present challenges to joint attention (i.e., shared focus of two people on the same object or event). When an infant physically explores objects while the grandparent watches on their screen, the infant may lack the ability or awareness to keep toys visible to grandparents, making it difficult for grandparents to comment on and to maintain the playful interaction. Conversely, when grandparents play with objects on their own side of the screen, infants' underdeveloped attentional capacity may make it challenging for them to remain focused on objects presented virtually (Oakes 2023), although infant abilities to visually track objects and people increases with age (Johnson et al. 1998; Oakes 2023). It is also more difficult for infants and grandparents to follow each other's gaze to objects on the other side of the screen: due to camera and screen placements, the angle of the on‐screen partner's gaze cannot be followed directly. Despite these challenges, prior studies have shown that joint attention can be established over video chat and its frequency increases with age and sensitivity of the adults involved (McClure et al. 2018; Myers et al. 2024).

As families embrace the unique attributes of the video chat format, they may creatively adapt their play to support infant engagement and attention. For instance, caregivers may respond to media errors by “pretending” that they received objects through the screen, turning the activity into pretend play. McClure and Barr (2017) reported that when an infant attempted to share raisins with her grandfather through the screen, the grandfather “took” the raisins and pretended to eat them. Parents and grandparents also encouraged children to engage in what would have been physical actions if they had occurred in person, including giving virtual hugs and kisses (Piper et al. 2023).

Both the person on screen and adults in the room with the child can play a role in these adaptations. For instance, although technical disruptions on video chat (e.g., a lost connection) can interrupt the flow of interactions, families in previous research (Piper et al. 2023) creatively adapted by modifying games and activities (e.g., turning a grandparent's disappearance due to the lost connection, and then coming back on screen, into “peek‐a‐boo”). McClure and Barr (2017) observed the adults on both sides of the screen working together: a mother tickling the child's toes for grandma while grandma sang “This Little Piggy” over video chat. Piper et al. (2023) reported other adaptations due to the adults' awareness of the limited view of the camera, such as grandparents changing the characteristic motions of the game “Head, Shoulders, Knees, and Toes” to body parts above the belly button, so that all motions would be visible on screen. Playful activities that have been observed on video chat differ by age, but include games such as peek‐a‐boo, show‐and‐tell, and singing songs (McClure and Barr 2017; Myers and McKenney 2021). Grandparents are also skilled at including questions about their grandchildren's behavior to indicate their interest and sustain children's attention during video chat (Ramirez et al. 2024) and they respond in positive, socially contingent ways that support higher engagement (McClure et al. 2018; Strouse et al. 2018; Troseth et al. 2018).

Families may also help children bridge between in‐person and video chat interactions by engaging in similar activities across both contexts, such as always waving goodbye or singing a favorite song on video chat they have frequently sung together in person. Theorists have argued that making explicit connections between similar real‐world and virtual experiences (e.g., to have the same adult playing the same game with you) are most useful during early childhood, when infants and toddlers have not yet mastered the representational and informative function of video images (e.g., Barr and Kirkorian 2023; Myers et al. 2017). Therefore, using familiar family games in the two kinds of sessions may also be a form of adaptation to the challenges of video chat.

Given the adaptations that families make to facilitate video chat play, virtual play may be unique in the duration, type, and amount of play that occurs. As children become more experienced with the adaptations that support positive video chat interactions, they may become more likely to engage positively and initiate video chat interactions. In addition, more advanced cognitive skills may enable infants to better navigate the challenges of screen‐based interactions (Myers et al. 2017), leading to video chat play interactions becoming more frequent, varied, successful, and infant‐initiated with age.

### 1.3 The Current Study

The present study is a pre‐registered secondary analysis of a longitudinal study of 47 infant‐parent‐grandparent triads. The original methodology describing data collection is also pre‐registered on OSF. This dataset leveraged the widespread use of video chat due to the separation of families during the COVID‐19 pandemic to examine how infants and grandparents engaged with one another during naturalistic video chat sessions and in‐person interactions. The present study examined two key research questions:

#### 1.3.1 RQ1. What are the Characteristics of Intergenerational Play on Video Chat?

##### 1.3.1.1 RQ1a. What Predicts Playful Activities That Occur Across the Screen (Between the Child and Remote Grandparent)?

We examined three measures of play: play bout frequency (number of playful interactions), play repertoire (number of different types of play) and proportion of time spent in play (out of the total length of the video chat session). We predicted that more frequent contact with grandparents on video chat (prior to/outside of the study) and older infant age would predict a higher frequency of play bouts, a larger play repertoire, and a greater proportion of time spent in across‐screen play during video chat sessions.

##### 1.3.1.2 RQ1b. What Predicts the Proportion of Attempted Play Bouts in Which the Infant Is Positively Engaged During Across‐Screen Play on Video Chat?

We predicted that a greater frequency of play bouts, greater play repertoire, and a greater proportion of time spent in across‐screen play would each predict a higher proportion of positive infant engagement with grandparents across the screen, accounting for infant age and frequency of infant‐grandparent video chats. Infant engagement is defined as the proportion of play bouts for which infants initiated the play interaction and/or responded with positive affect or action to their grandparent's action (e.g., infant giggles/smiles in response to Grandma making a silly noise).

##### 1.3.1.3 RQ1c. What Predicts Infant Initiation of Across‐Screen Play on Video Chat?

We predicted that a greater frequency of play bouts and a greater proportion of play would be associated with a greater proportion of across‐screen play interactions initiated by infants, controlling for infant age and frequency of infant‐grandparent video chats. We did not have a directional hypothesis for the association between play repertoire and the proportion of across screen interactions that were initiated by infants.

#### 1.3.2 RQ2. Does Play on Video Chat Differ From Play In‐Person?

We examined whether the frequency of play bouts, play repertoire, the proportion of time spent in playful activities, and the proportion of bouts in which the infant positively engaged differed between video chat and in‐person sessions. Given the challenges of interactions on video chat, we expected infants and grandparents to spend more time playing and to have more instances of positive engagement with infants when interacting in person compared to video chat. We were also interested in overlap, or the degree to which families participated in similar activities on video chat and in person.

## 2 Method

This study follows the methods of Roche et al. (2022), Piper et al. (2023), and Myers et al. (2024) and therefore portions of the method repeat from those papers.

### 2.1 Participants

Participants of the original study were 50 parent‐grandparent‐infant triads with infants born between September 2019 and September 2020 who lived in the United States and Canada. For the current study, one family from the original study was excluded because of poor video quality and two additional families were excluded for having fewer than 2 video chat sessions; therefore, the final analysis included data from 47 parent‐grandparent‐infant triads (29 male infants, 18 females). Families recorded three video chat sessions approximately every 2 months: infants' age at initial session was *M* = 9.66 months (SD = 2.59, range 5–15 months), at the second session *M* = 12.61 months (SD = 2.86, range 6–19 months), and at the third session *M* = 14.79 months (SD = 2.74, range 9–20 months). In addition to the video chat recordings, 40 of the 47 families recorded an in‐person session. 30 of these recordings were at least five minutes long and captured playful activities between the grandparent and infant; only these families were included in our in‐person analysis. The average infant age at the in‐person session was 11.74 months (SD = 3.56, range 4–21 months). The majority of parents were English‐speaking (one family spoke Turkish). Parents and grandparents predominantly self‐identified as White; one grandparent identified as African American, and one parent as mixed race. In addition, two grandparents and five parents identified as Latino/Hispanic. Parents ranged from 24 to 41 years of age (*M* = 32.8; SD *=* 4.02) and were mostly mothers (5 fathers and one non‐binary parent). Grandparents (all grandmothers) ranged from 47 to 76 years (*M* = 62; SD *=* 6.61). Parents reported education levels of high school (2%), 2‐year degree (6%), 4‐year degree (38%), advanced degrees (54%), whereas grandparents reported less than high school (2%), high school/GED (8%), 2‐year degree (20%), 4‐year degree (28%), advanced degrees (42%). Participants were recruited through Research Match, Children Helping Science, preexisting lab databases, community partnership databases, and social media advertising.

### 2.2 Materials

Consents and survey data were collected over Qualtrics and REDCap. Video chat recordings were conducted via Zoom and were automatically uploaded to the cloud upon completion of each session. Families used laptops, desktop computers, and cell phones for video chat recordings. In‐person recordings were conducted in a similar fashion, but some families manually e‐mailed video files from their phone rather than using a single‐user Zoom link to record the in‐person session.

### 2.3 Design

Participants recorded three family video chats spaced approximately 2 months apart, starting about 1 month after they were recruited for and consented to the study. Families were also asked to record an in‐person meeting that occurred after they were recruited for the study.

### 2.4 Procedure

Data collection lasted from August 2020 to August 2021. After consenting to participate, surveys were collected from both parents and grandparents on Qualtrics, from which we report participant demographics. Prior to each session, participants completed a survey on REDCap, from which we report the frequency of video chat contact between the infant and grandparent that occurred in the 2 months prior to that recorded video chat session. Before a family's first video chat, the parent or grandparent met with a researcher who provided general instructions for the study. Participants were sent a Zoom link via email after the meeting, which they used to record their later Zoom and in‐person meetings without the researcher's presence or involvement. Participants were compensated for their time and received a $5 e‐gift card for each survey completed and a $10 e‐gift card for each video recording.

Participants were instructed to record at least 15 min of a video chat with all 3 participants present. Other people were often included in the video chat recording, with about 4 participants on average (*M* = 3.99 people, SD = 0.97). Participants were instructed to record at least 5 min of their in‐person session with the grandparent and infant. Sometimes the parent recorded but did not directly participate in the in‐person session. There were approximately four participants on average in the in‐person recordings (*M* = 3.81 people; SD = 1.04), and the number of people in the recordings and the video chats was not significantly different, *t* (63.99) = 0.97, *p* = 0.333.

Video chats and in‐person sessions varied in duration. Up to 25 min were used in analyses that included only video chat data (RQ1). The average length of video chat recording after truncation of videos over 25 min was 18.18 min (SD = 5.63 min). Videos of in‐person sessions were shorter than video chats (*M* = 9.59 min: SD = 5.19 min). Therefore, for comparisons of video chat and in‐person sessions (RQ2), both kinds of sessions were truncated to 5 min. Variability in when during the study families recorded their in‐person sessions was accounted for in our analyses by comparing the in‐person session to the video chat session that occurred closest in time.

### 2.5 Coding of Playful Activities

Video recordings were reviewed by trained coders who identified instances of playful activities. Codes for playful activities were determined in two ways. First, codes were generated using results of a survey that had been sent out to parents and grandparents in prior research, asking them to indicate activities they engaged in when video chatting with their infant (Myers and McKenney 2021). These activities included singing, dancing, playing with a toy, peek‐a‐boo, waving, clapping, imitation games, show‐and‐tell, hide‐and‐seek, and joint pretend play (engagement in a social activity that involved imaginary or impossible objects, actions, or ideas, such as a child trying to pass a basketball through a screen). A second way codes were determined was through initial coding of the first few video chat recordings, which allowed us to capture additional playful activities not included in the survey. A code for silly noises was added based on this review. Playful activities were coded as *other* if they did not fit into any of the identified categories; some examples include making silly faces, banging musical instruments, infant and grandma drawing with one another on both sides of the screen. Only playful activities that involved both the infant and grandparent were coded, although other family members were also sometimes involved.

Each playful activity was coded for the type of play, duration, who was visible on the recording, and whether the infant was positively engaged during the activity. For the video chat sessions, we also coded the role of each individual (as the initiator of the play bout, the recipient, or the assistant). Coders were trained to criterion (Kappa value of 0.70) and 20% of videos were double‐coded, achieving Kappas of above 0.80 for all playful activity codes.

### 2.6 Summary Variables

#### 2.6.1 Frequency of Infant‐Grandparent Video Chat

Parents and grandparents self‐reported the frequency of infant‐grandparent video chat interactions during the previous 2 months in a survey completed before each session. For the first session, this measure of video chat frequency covered the 2 months prior to the study. For the remaining sessions, this item roughly measured the frequency of video chat between sessions. We calculated the average of the parent's and grandparent's reports for each family and session. Mean scores ranged from: Every day (4), A few times a week (3), A few times a month (2), Less than once a month (1), and Never (0).

#### 2.6.2 Frequency of Play Bouts

**FIGURE 1:**
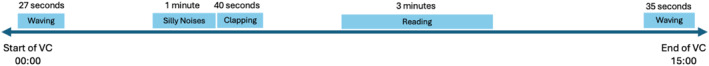
Depiction of a sample video chat session.

Play bout frequency was the total number of instances of playful activities that triads engaged in during each session. As an illustrative example, Figure 1 depicts the play bouts of one family with a frequency of 5.

#### 2.6.3 Play Repertoire

The total number of *different types* of playful activities that triads engaged in during each session was the play repertoire. When multiple types of play happened at the same time (e.g., if singing and dancing occurred simultaneously), both were added to the family's play repertoire as distinct activity types. The family depicted in Figure 1 had a play repertoire of four activities (i.e., waving at the beginning and end of the session counted as one type of activity).

#### 2.6.4 Proportion of Play

The proportion of time spent engaged in playful activities during each session was calculated as the proportion of session time spent in play between the infant and grandparent. The proportion of play for the family in Figure 1 was 0.38 (342 total playful seconds divided by 900 s total time). Time when the parent and infant played together without the grandparent, and when the infant played on their own, was not included in the proportion of play.

#### 2.6.5 Proportion of Infant Engagement

Positive infant engagement was coded as present during a play bout if the infant exhibited positive affect, an engaged response, or participation in the activity, even if they later lost interest. Conversely, infant engagement was coded as absent during a play bout if the infant consistently demonstrated negative affect, a lack of interest, or signs of irritability throughout the activity. Proportion of infant engagement therefore was the proportion of play bouts in which the infant was positively engaged (out of total play bouts) across the session.

#### 2.6.6 Proportion of Infant Initiation

Infant initiation was calculated as the proportion of play bouts on video chat during which the first action in the play sequence was performed by the infant, such as the infant picking up a toy and showing it to Grandma.

#### 2.6.7 Overlap With In‐Person Play Scores

To assess the similarity of in‐person and video chat play, overlap scores were computed for each family using a similarity index that captured whether the types of play that infants engaged in during the in‐person session also occurred during the video chat session that was closest in date to the in‐person session. This included play that was initiated by the infant, parent, or grandparent. An overlap score (proportion) was calculated by dividing the similarity index by the total number of play activities observed in the in‐person session. For example, if during the in‐person session the infant engaged in peek‐a‐boo, singing, dancing, and silly noises (repertoire of 4) and two of those activities (singing and dancing) also occurred in their video chat, the family would have a similarity index of 2 and an overlap score of 0.5. The overlap score did not take into account how many additional activities the family did during the video chat, but those activities were captured in the family's video chat repertoire score.

## 3 Results

### 3.1 Descriptive Statistics

Table 1 shows descriptive statistics for the frequency, repertoire, and proportion of play during the video chat and in‐person sessions as a function of infant age. The column headed “Video chat (full session)” reports averages for the three video chat sessions. The two right columns present a comparison of data from the first 5 min of the video chat session and the in‐person session that were closest in time. Although play only occurred during 13–22% of the session, most infants engaged in multiple play bouts with varied activities. Infants engaged positively in a majority of the play bouts both on video chat and in person. The proportion of play bouts initiated by the infant on video chat was higher for older than younger infants, but still relatively low.

**TABLE 1:** Descriptive statistics for video chat and in‐person sessions as a function of mean infant age.

		First 5 min	
	Video chat (full session)	Video chat	In‐person
Session duration minutes (SD)			
Younger infants	18.38 (5.26)	5.00 (0.00)	5.00 (0.00)
Older infants	17.95 (5.96)	5.00 (0.00)	5.00 (0.00)
Play frequency (SD)			
Younger infants	7.65 (4.56)	3.62 (3.07)	3.46 (2.44)
Older infants	9.41 (5.66)	3.80 (2.57)	3.40 (2.67)
Play repertoire (SD)			
Younger infants	3.52 (1.67)	2.38 (1.45)	2.00 (0.91)
Older infants	4.09 (1.81)	2.60 (1.26)	2.00 (0.67)
Play proportion (SD)			
Younger infants	0.11 (0.08)	0.13 (0.09)	0.19 (0.19)
Older infants	0.12 (0.08)	0.18 (0.17)	0.22 (0.18)
Infant engagement proportion (SD)			
Younger infants	0.73 (0.27)	0.89 (0.16)	0.94 (0.15)
Older infants	0.83 (0.18)	0.87 (0.31)	0.89 (0.21)
Infant initiation proportion (SD)			
Younger infants	0.13 (0.21)	0.10 (0.21)	N/A
Older infants	0.21 (0.25)	0.19 (0.33)	N/A

*Note:* Younger refers to infants below the mean age of 12.33 months (*n* = 71 data points for VC across 3 video chat sessions; *n* = 13 data points for the first 5 min of the closest VC to IP; *n* = 13 data points for IP). Older refers to infants above the mean age of 12.33 months (*n* = 64 data points for VC across 3 video chat sessions; *n* = 10 data points for the first 5 min of the closest VC to IP; *n* = 10 data points for IP).

**FIGURE 2:**
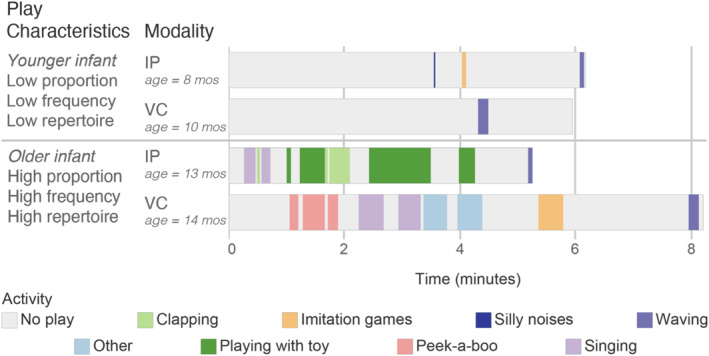
Play Characteristics for an Infant above and below Mean Infant Age. Each pair of rows represents one family's in‐person (IP) recording and the video chat (VC) that occurred closest in time to the in‐person recording. Top panel: Family with a younger infant, whose age was below the mean (*M* = 12.33 months). Bottom panel: Family with an older infant.

Descriptive analyses also revealed variability in play type, frequency, repertoire, and proportion between families, across modalities, and as a function of infant age. To illustrate the variability in play, two families are presented in Figure 2. The first family with a younger infant (top panel) displayed a low frequency of play bouts, a low repertoire of play, and a low proportion of play in person and on video chat. The family completed three activities in person and one activity on video chat, both for short bursts of time with high overlap between activities. The second family with an older infant (bottom panel) displayed a high frequency of play bouts, a high repertoire of play activities, and a high proportion of play in both modalities. As highlighted by the blocks of color, this family took part in many different types of play both in person and on video chat. Play bouts lasted for longer blocks of time and involved switching between different activities, but there was less overlap in activities than the first family. Play activities for in‐person and video chat sessions for all families sorted by infant age can be found in Supporting Information S1: Figure S1 in the supplemental materials.

**FIGURE 3:**
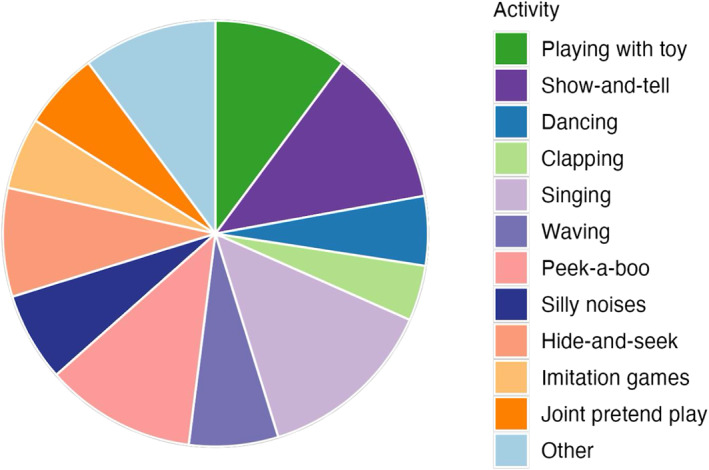
Average proportion of video chat play spent in each activity.

Figure 3 shows the average proportion of play spent in each activity (e.g., dancing, peek‐a‐boo). The activities spanned multiple categories described in the play literature, such as playing with toys (object play); waving, peek‐a‐boo, and hide‐and‐seek (social play); and joint pretend play. All of the activities reported required some level of social interaction, since we only coded play when it involved both the infant and grandparent. We had no predictions about, and made no comparisons between, activity types in the remaining analyses.

### 3.2 RQ1 Data Analysis Plan

To address RQ1 regarding the predictors of intergenerational play on video chat we fit growth models to predict (a) across‐screen play measures (frequency, repertoire, proportion), (b) infant engagement, and (c) infant initiation of across‐screen play. Since families had multiple sessions across the study, we first estimated unconditional mixed effects linear models to assess clustering by family ID. Mixed effects models were estimated using the *lme4* package in *R* (Bates et al. 2025; R Core Team 2021). We then assessed linearity in the associations between infant age and each outcome variable. Upon visual inspection, a quadratic transformation of infant age did not improve linearity in the associations with each of the play characteristics, so only the untransformed age variable was used. We also assessed for the inclusion of random slopes and intercepts for infant age with likelihood ratio tests between unconditional models and those that included random slopes and intercepts. Models including random slopes for infant age either did not converge or did not improve fit; therefore, only fixed slopes for infant age were retained in the reported models. We assessed collinearity among the predictor variables in each model reported below. Correlations were moderate, but not problematic (Variance Inflation Factor range: 1.00–1.88; Sheather 2009).

Our pre‐registered analysis plan included a calculated variable, called video chat experience, representing an interaction between infant age and concurrent infant‐grandparent video chat frequency. While building the final models, we updated our plan by replacing the video chat experience variable with an interaction between age and video chat frequency to also assess associations between the variables and our outcomes. However, this interaction was not significantly associated with any of the outcomes. To simplify reporting of our results, the models including this interaction can be found in Table S1 in the supplemental materials.

Characteristics of play used for this research question included *frequency* (number of individual bouts of play with the grandparent across the session; *M* = 8.48, SD = 5.17), *repertoire* (number of unique shared play activities in each session; *M* = 3.79 SD = 1.75), *proportion* of total session time engaged in across‐screen play (*M* = 0.11, SD = 0.08), *infant engagement* (proportion of playful activities in which the infant was positively engaged; *M* = 0.41, SD = 0.34), and *infant initiation* (proportion of playful activities initiated by the infant; *M* = 0.78, SD = 0.24). At the session level, we included two predictors in all models: infant age and frequency of video chat over the prior 2 months, or how often grandparents and parents self‐reported video chatting with the infant outside of the study itself. We also controlled for session duration in all models except for the one predicting play proportion, because this variable already accounted for the video chat's duration. All significant coefficients are presented while holding the other variables constant.

### 3.3 RQ1a. What Predicts Across‐Screen Play on Video Chat?

For play frequency, 30% of the variance in frequency scores could be attributed to differences between families (Intraclass Correlation Coefficient [ICC] = 0.299). Clustering by family accounted for 39% of the variance in play repertoire (ICC = 0.385) and 30% of the variance in play proportion (ICC = 0.303). Typical ICC values in social science studies fall between 0.05 and 0.25 because of the consistency of human behavior (Snijders and Bosker 2012). Our use of multilevel modeling, which accounts for repeated measures of the same individuals, is supported by the amount of clustering observed in the data, which reflects that families were consistent with their own play behaviors across sessions.

#### 3.3.1 Play Frequency

Infant age (*b* = 0.26, *p* = 0.050) was marginally related to increased play bout frequency (Table 2). A 2.6‐month increase in infant age was associated with one more playful activity during a session. Video chat duration (*b* = 0.19, *p* = 0.018) was also significantly associated with increased play bout frequency. This estimate translates into an additional play bout for each 5‐min increase in video chat duration. Video chat frequency was marginally significantly associated with play bout frequency (*b* = 0.99, *p* = 0.070).

**TABLE 2:** Mixed effects models results for play frequency, play repertoire, and play proportion.

	Play frequency				Play repertoire				Play proportion			
	Estimate	SE	*t*	*p*	Estimate	SE	*t*	*p*	Estimate	SE	*t*	*p*
(Intercept)	−0.56	2.41	−0.23	0.818	0.39	0.79	0.49	0.626	0.10**	0.03	2.97	0.004
Infant age (months)	0.26+	0.13	1.98	0.050	0.09*	0.04	2.26	0.026	0.00	0.00	−0.12	0.906
Video chat frequency	0.99+	0.54	1.83	0.070	0.34+	0.19	1.81	0.073	0.01	0.01	0.77	0.442
Session duration	0.19*	0.08	2.39	0.018	0.08**	0.03	3.00	0.003	—	—	—	—
SD (intercept)	2.35				1.05				0.05			
SD (Obs.)	4.23				1.25				0.07			
Num.Obs.	132				132				132			
*R* ^*2*^ marg.	0.109				0.132				0.006			
*R* ^*2*^ cond.	0.320				0.492				0.316			
AIC	800.8				509.8				−259.8			
ICC	0.2				0.4				0.3			
RMSE	3.87				1.10				0.06			

*Note:* +*p* < 0.1, *p < 0.05, **p < 0.01, ***p < 0.001.

Abbreviations: ICC = interclass correlation coefficient, RMSE = root mean square error.

#### 3.3.2 Play Repertoire

Infant age (*b* = 0.09, *p* = 0.026) and video chat duration (*b* = 0.08, *p* = 0.003) were associated with play repertoire (Table 2). A 9‐month increase in infant age was associated with one more activity in the play repertoire. A 12.5‐min increase in video chat duration was associated with one more activity in the play repertoire. Video chat frequency was also marginally significantly associated with play repertoire (*b* = 0.34, *p* = 0.073).

#### 3.3.3 Play Proportion

Infant age and frequency of video chat were not significantly associated with play proportion (Table 2). Session duration did not apply here because proportion already accounted for total duration.

### 3.4 RQ1b. What Predicts the Proportion of Attempted Play Bouts in Which the Infant Is Positively Engaged During Across‐Screen Play on Video Chat?

The proportion of attempted play bouts in which the infants were positively engaged during a session was high across the sample (*M* = 0.77, SD = 0.24; see Figure S2 for engagement across sessions per family). Unconditional multilevel models predicting infant engagement suffered from singularity when clustering by family because the random effect variance was estimated to be essentially zero. We estimated pooled OLS regressions for engagement with cluster‐robust standard errors to account for the clustered nature of the data (Wooldridge 2010). Standard errors were clustered by family to account for repeated measures within the same families.

We deviated from our pre‐registered analysis plan by including all three play measures (frequency, repertoire, proportion) in our final model. This new approach had the advantage of being more parsimonious. Infant age was associated with infant engagement such that older infants had a higher proportion of play bouts with positive infant engagement compared to younger infants (*b* = 0.02, *p* = 0.007). Infant engagement in play was not related to play proportion, repertoire, or video chat duration (Table 3).

**TABLE 3:** OLS regression results for positive infant engagement.

	Estimate	SE	*t*	*p*
(Intercept)	0.54***	0.15	3.72	< 0.001
Play frequency	0.01	0.00	1.31	0.192
Play repertoire	0.01	0.01	0.53	0.594
Play proportion	−0.31	0.38	−0.82	0.416
Infant age (months)	0.02**	0.01	2.73	0.007
Video chat frequency	−0.01	0.02	−0.26	0.796
Session duration	0.00	0.01	−0.26	0.798
Num.Obs.	132			
*R* ^*2*^	0.06			

^**^ *p* < 0.01.

^***^ *p* < 0.001.

### 3.5 RQ1c. What Predicts Infant Initiation of Across‐Screen Play on Video Chat?

Clustering video chat sessions within families accounted for 25% of the variance in infant initiation (ICC = 0.248). Once again, we deviated from our pre‐registered analysis plan by including all three play measures in our final model (which was more parsimonious), rather than running separate models for each. Infant age was significantly associated with initiation of play such that older infants initiated more bouts of play per session (*b* = 0.02, *p* < 0.001). Play proportion, play frequency, play repertoire, frequency of infant‐grandparent video chat, and video chat duration were not related to infant initiation (Table 4).

**TABLE 4:** Multilevel model results for infant initiation of play.

	Estimate	SE	*t*	*p*
(Intercept)	0.04	0.13	0.31	0.754
Play frequency	0.00	0.01	0.15	0.881
Play repertoire	−0.01	0.01	−0.58	0.561
Play proportion	−0.26	0.32	−0.81	0.421
Infant age (months)	0.02***	0.01	3.82	< 0.001
Video chat frequency	−0.04	0.02	−1.54	0.127
Session duration	0.00	0.00	−0.04	0.966
SD (intercept familynum)	0.11			
SD (Observations)	0.19			
Num.Obs.	132			
*R* ^*2*^ marg.	0.131			
*R* ^*2*^ cond.	0.337			
AIC	30.6			
ICC	0.2			
RMSE	0.18			

Abbreviations: AIC = akaike information criterion, ICC = interclass correlation coefficient, RMSE = Root mean square error.

^***^ *p* < 0.001.

### 3.6 RQ2. Does Play on Video Chat Differ From Play In‐Person?

We examined differences between the in‐person and video chat sessions using the same outcomes that were examined only in the video chat context for RQ 1a and 1b: play *frequency* (number of individual bouts of play across the session), play *repertoire* (number of unique play activities in each session), play *proportion* (time engaged in play with grandparent), and infant *engagement*. In‐person recordings that were at least 5 min long were submitted by 31 families. There was no play in the first 5 min of four in‐person recordings and 4 video chat recordings, resulting in data from 6 families being dropped from this analysis. A one‐way MANOVA was run including data from the remaining 23 families comparing play frequency, proportion, repertoire and engagement as a function of play context (in person vs. video chat). There were no differences between in‐person and video chat play (Pillai's trace = 0.17, *F* (4, 45) = 2.34, *p* = 0.070).

**FIGURE 4:**
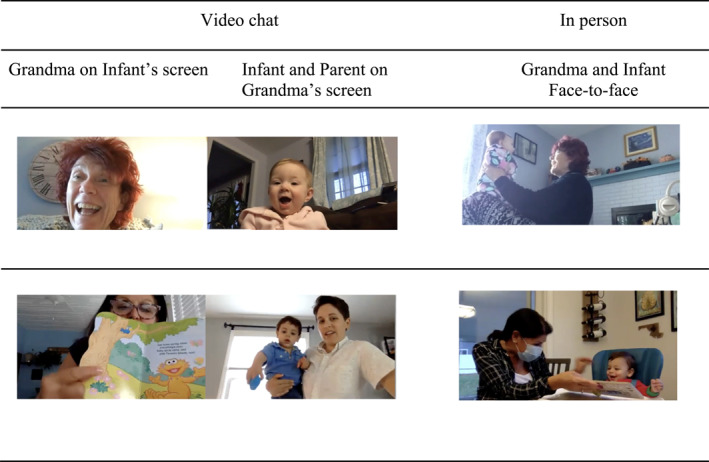
Playful interactions between the same grandparent‐infant dyads on video chat and in person.

To further explore similarity between in‐person and video chat play we calculated an overlap score that represented the overlap in play activities from in‐person interactions to the closest video chat session. The highest possible score (1.0) would indicate that 100% of the play activities that families engaged in during the in‐person session were also observed during their closest‐in‐time video chat interaction. Overlap scores were calculated for families with an in‐person recording that was at least 5 min long (*n* = 30). The mean amount of play overlap for families was *M* = 0.38 (SD = 0.34). Overlap scores ranged from 0 to 1, indicating that some families engaged in none of the same activities in person and on video chat, and some families engaged in all of the in‐person activities during their video chat session. Figure 4 depicts illustrative playful interactions between grandparents and infants that were similar on video chat and in person with the same dyad.

## 4 Discussion

The present study explored the amount (frequency and proportion of play bouts) and range (repertoire) of playful activities that occur during intergenerational play on video chat, and whether these aspects of play predicted infant engagement in and initiations of play. We then examined whether play frequency, repertoire, proportion, or engagement differed between in‐person and video‐chat contexts and explored how frequently play activities overlapped in the two contexts. Infant age emerged as the strongest predictor of the play outcomes during video chat across models. Infants participated in a range of playful activities in both contexts but displayed a larger repertoire on video chat than in person, with some overlap in the types of activities they engaged in across contexts. The results from this study offer insight into how infants and grandparents play over video chat, and how these interactions change based on infant age and modality.

### 4.1 Playful Activities on Video Chat

Infants engaged in multiple short play bouts during their video chat sessions, similar to the frequent, short bursts of play that have been observed in non‐video chat contexts (Adolph and Robinson 2015; Herzberg et al. 2022; Kannass et al. 2006). These play bouts spanned many different types of play, including object play, social play, and pretend play. Although infant initiations of play were rare, infant engagement in play activities was high, illustrating that adult‐initiated play bouts were often received positively by infants.

As hypothesized, we found that infant age significantly predicted play repertoire. That is, older infants took part in a greater variety of activities than younger infants did. Age also significantly predicted infant engagement and initiation of play bouts, and marginally predicted play frequency. These developmental changes in video chat play could be the result of infant cognitive or social development, as infants become able to engage in more cognitively complex types of play as they get older (Lillard et al. 2013; Piaget 1962). Beyond changes in types of play, cognitive development may also support infants in becoming better able to navigate aspects of the video chat context (Barr and Kirkorian 2023) such as attending to on‐screen objects or following gaze across the screen, which would support a greater repertoire of activities. Age trends may in part reflect overall video chat experience accumulated both with grandparents and other video chat partners. It is also possible that age‐related differences are a result of changes in adult behavior, as adults interact with infants of different ages in different ways.

Contrary to our prediction, the frequency of infant‐grandparent video chat over the prior few months did not significantly predict any of our outcomes after controlling for age, although it was marginally related to play frequency and repertoire. The duration of the video chat was a significant predictor for both of those outcomes, indicating that longer sessions had more play bouts and different types of play. The interaction between the frequency of video chat and the age of the infant also did not significantly predict any outcomes, and related analyses were therefore moved to the supplemental materials. Frequent video chat sessions (even if short in duration) may normalize the video chat context for infants and facilitate their engagement with the on‐screen partners. Responsive and supportive co‐viewers during video chat sessions may model successful video chat interactions (McClure et al. 2018; Myers et al. 2018). One limitation was that the video chat frequency question in the current study was a one‐item Likert scale; future researchers should collect more comprehensive measures of video chat experience to disentangle its effects from age‐related trends.

Also contrary to our hypotheses, infant age did not predict the proportion of time spent playing on video chat and only marginally predicted play bout frequency. This suggests that despite challenges to engaging in play on video chat, especially for young infants, families are engaging in similar amounts of play with younger and older infants. Prior studies (McClure and Barr 2017; Piper et al. 2023) have shown that families adapt common play activities to make them more appropriate for the video chat context, and our results suggest that families may be doing these adaptations across the age range studied here, although age‐related differences in infant engagement suggest that adaptations may be more successful as infants get older.

Finally, play bouts, play repertoire, and the proportion of time spent in play did not predict infant engagement or infant initiations of play. Instead, only age predicted these outcomes. Although introducing a wider repertoire of play activities may be one strategy for successfully re‐engaging infants with waning attention, our findings suggest that frequent variation of activities is not a universal strategy for engaging infants.

### 4.2 Comparing Playful Activities Across Video Chat and In‐Person Sessions

There was no difference between the proportion of play bouts, play repertoire, the proportion of time spent in play, or the proportion of infant engagement across modalities. Infant engagement was near ceiling in both contexts, indicating an overall positive experience in both play contexts. Overlap scores may provide insight into how families use their knowledge of what has worked in person to inform their play on video chat. Our results indicate that families chose to do some of the same activities both in person and during video chat play, but many activities were unique to each context.

To our knowledge, ours is the first work to examine infants' naturalistic play during video chat, and we have shown that there are striking similarities in play between the two contexts. Although video chat presents social, attentional and cognitive challenges, we have shown that infants and families can be remarkably adaptive to the virtual context. The family connection behind the interactions in both contexts remained relatively similar‐grandparents, parents, and infants still laughed, still smiled, and still played with one another over a screen, the way they did when face to face. For example, one participating grandmother had two llama dolls (“Lulu” and “Lenny”) that she used heavily in all the video chat sessions with her grandson. In an email she expressed, “Lulu and Lenny have been fun to incorporate into our video chats, and I do like to think it's helped *B* “connect the dots” to us! Now I'm counting down the days until my visit in August!” This grandmother had repeatedly used the same toys in an attempt to help her grandson build continuity between their video chat interactions and real‐life visits. As this family's experience demonstrates, we provide additional evidence of families' remarkable creativity as they adapted play to support infant engagement (McClure and Barr 2017). These efforts are likely successful because explicit overlap between real‐world and virtual experiences helps infants make the connection between the two contexts at an age when they do not fully understand the representational aspects of video images (Barr and Kirkorian 2023; Troseth 2010). Our results mirror past work on naturalistic play in person (Kannass et al. 2006; Adolph and Robinson 2015) and extend those results to the video chat context.

Grandparent involvement in a child's life has been linked with both direct (child development) and indirect (parent support) benefits for families. However, one challenge for grandparent involvement is that 36% of grandparents live 30 or more miles away from their grandchildren (Healy and Dunifon 2025). Also, isolation events like COVID‐19, disruption to air travel, and tightened budgets due to recessions may impact the opportunities for in‐person contact. Our research indicates that video chat can be a powerful tool for intergenerational play for families, which potentially may support emotional closeness for all parties as well as developmental benefits to children. In a recent survey, 41% of parents reported feeling so stressed that they cannot function, and 48% reported feeling that their stress is completely overwhelming, suggesting the potential value of leveraging grandparent‐grandchild relationships to support families (Office of the Surgeon General (OSG) 2024). The current study indicates that very young grandchildren and their grandparents were able to playfully interact with one another over video chat and suggests that playful interactions on video chat and in person may provide an additional positive context for development and family support.

### 4.3 Limitations and Future Directions

There are several limitations in the present study. First, the sample size was small and lacked adult gender diversity, with the majority of participants identifying as mothers and grandmothers. This limits the generalizability of the results to the broader population of caregivers, particularly fathers and grandfathers. Prior research indicates that both adult and infant gender may influence grand parenting dynamics (Mueller and Elder 2003). For instance, men are more likely to engage in physical, rough‐and‐tumble play, especially with boys (St George and Freeman 2017), potentially making their transition to playing over video chat more difficult. Future work should examine gender differences in play, as more male caregivers and grandparents may reveal different types of interaction in virtual contexts.

Our sample also lacked racial, cultural, educational, and socioeconomic diversity; it reflected the sample of individuals who had the time and resources to participate in this study during the peak of the COVID‐19 pandemic. This is especially important given the disproportionate impact of the COVID‐19 pandemic on families of color and those from lower socioeconomic status (Karpman et al. 2020). Many low‐income families have inconsistent access to the internet, known as “underconnectivity” (Katz et al. 2017). This issue disrupted the use of technology for such families and may have impeded their ability to participate in the study or to regularly engage in video chat‐based interactions more broadly. Future research should develop interventions for low‐resourced families that are geographically separated due to work, migration, incarceration or military service. These interventions could examine whether reducing under‐connectivity and providing well‐designed virtual play interventions supports positive video chat experiences and subsequently enhances intergenerational connections.

This study was also semi‐naturalistic, meaning there was no experimental manipulation, and participants were provided with very little guidance on how to interact on video chat. While semi‐naturalistic studies have a wide variety of benefits, particularly high ecological validity, this design also reduces the ability to draw causal conclusions or control potential confounds. For instance, due to various ethical considerations, we did not tell grandparents when to meet their grandchildren in person. As a result, the timing of in‐person contact varied widely across participants. Due to a lack of any play during the first 5 min of their video chat or in‐person sessions, we were unable to make cross‐modality comparisons for some of our families. Future research should incorporate experimental design to examine whether different types of play facilitate engagement, or whether play duration is an important consideration during video chat, as infants may get bored or become less engaged if play goes on too long, especially on video chat.

Another potential study limitation is that parents may have been differentially involved in the in‐person and video chat interactions. Despite these limitations, the findings still provide insights into how infants, caregivers, and grandparents engage and play over video chat. The limitations also highlight important directions for future research, particularly regarding the role of gender and digital equity in understanding and shaping remote interactions.

### 4.4 Implications

While further investigation is needed, this research provides insight into how grandparents and very young grandchildren interact with one another, both in person and on video chat. Families took part in many different kinds of activities on video chat, possibly trying different kinds of play or adaptations to determine which best supported engagement. Additionally, future research should examine whether continuity in activities across modalities plays an important role in infant engagement and helps facilitate interactions in both virtual and in‐person contexts.

It will also be important for future research to explore potential relative benefits of different types of playful activities for supporting intergenerational play on video chat. Specifically, given that in‐person play is often separated into free play, guided play, and games (Zosh et al. 2018), future research should examine if specific playful activities provide more opportunity for infant engagement versus other types.

Infant age was the largest predictor of infant engagement and initiation of play on video chat, which is important for parents and grandparents to take into account while on video chat with their infants. Even the youngest infants engaged in play with their grandparents on video chat and for a similar proportion of the session; however, older infants engaged in a greater variety of types of play, possibly reflecting growth in overcoming the cognitive challenges that could make some types of play on video chat difficult.

### 4.5 Conclusion

The goal of this research was to compare in‐person and video chat play and to gain a better understanding of how infants and grandparents engage in play during video chat. Infants engaged in a range of playful activities across both contexts, with age emerging as the strongest predictor across outcomes. Families may need to make adaptations to how they would play in person due to the unique attributes and challenges of the video chat context, and our study shows that families are successfully doing so. Our findings suggest that video chat may serve as a useful technology to strengthen intergenerational connections by capitalizing on play‐based interactions when family members are separated from each other, potentially opening up new avenues for family support.

## Author Contributions

**Lucinda I. Neely:** conceptualization, methodology, investigation, formal analysis, visualization, writing – original draft, writing – review and editing. **Douglas J. Piper:** conceptualization, methodology, investigation, formal analysis, supervision, visualization, writing – review and editing. **Lauren J. Myers:** conceptualization, methodology, investigation, supervision, writing – review and editing. **Jennifer M. Zosh:** investigation, supervision, writing – review and editing. **Gabrielle A. Strouse:** investigation, formal analysis, supervision, writing – review and editing. **Georgene L. Troseth:** investigation, supervision, writing – review and editing. **Rachel Barr:** methodology, investigation, supervision, writing – review and editing.

## Funding

This study was supported by the AARP and Lafayette College.

## Ethics Statement

The project was reviewed and approved by Georgetown University as the IRB of record (STUDY00002521). The study was approved by the institutional review board at the authors' institution.

## Conflicts of Interest

The authors declare no conflicts of interest.

## Supporting information


Supporting Information S1

## Data Availability

Raw data and analysis scripts are available via OSF here: OSF_link.
