# Supplementary material for: In the Living Room and Across the Screen: Intergenerational Play Between Infants and Grandparents
Source: Infancy. 2026 Jan 11;31(1):e70065. doi: 10.1111/infa.70065 (PMC12790811; doi:10.1111/infa.70065)
Supplement: Supplementary file 1 — Supporting Information S1 [file INFA-31-0-s001.docx]

**Figure S1**

*Play Activities for In-Person Sessions and Closest Video Chat Sessions Sorted by Age*


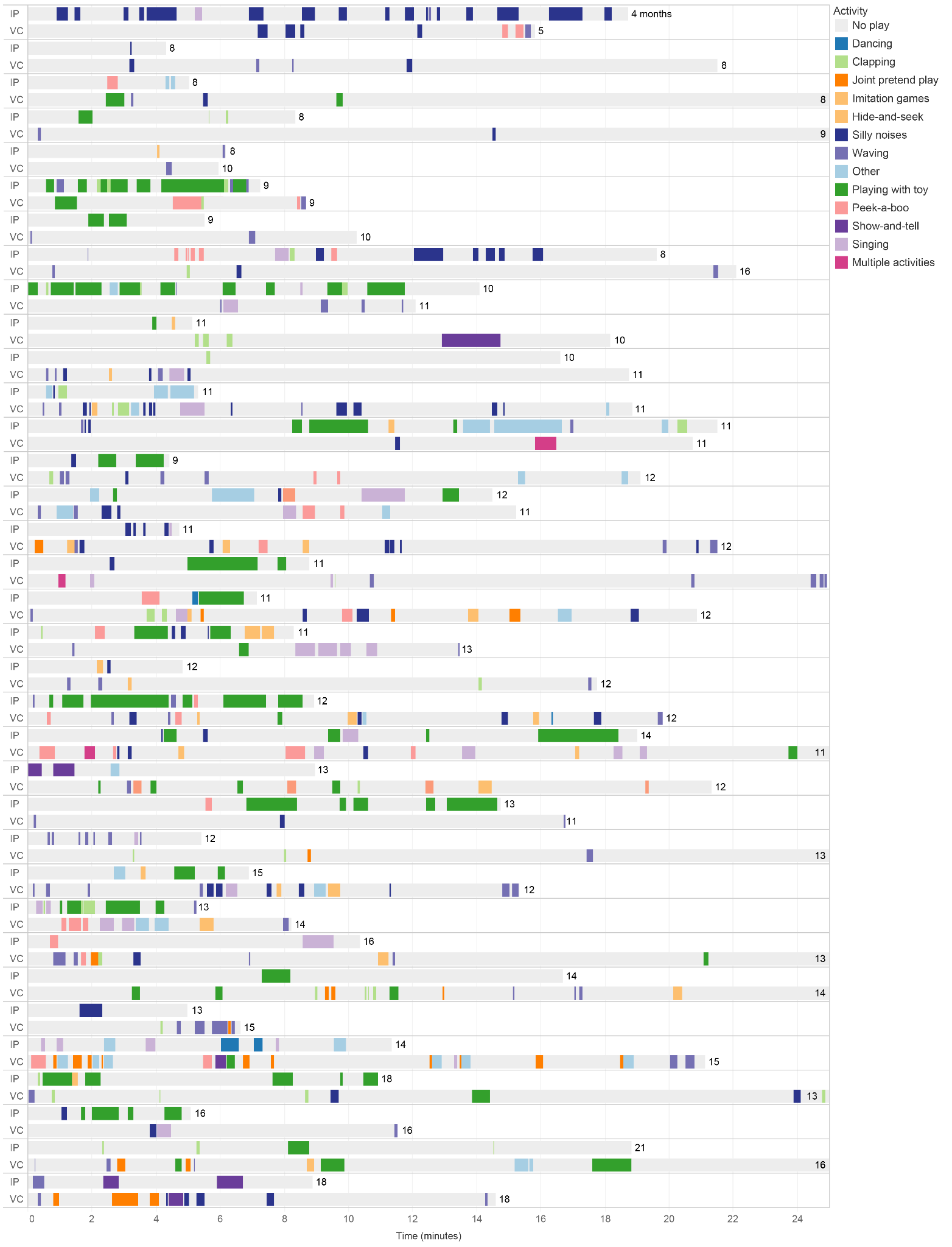


*Note.* Each in person (IP) and video chat (VC) pair represents one family separated by the dark gray lines. Families are sorted by mean infant age between both sessions from youngest (top) to oldest (bottom). In-person sessions took place within three months of the nearest video chat. Recordings were truncated to the first 25 minutes when applicable.

**Figure S2**

Proportion of Successful Infant Engagement across Sessions per Family


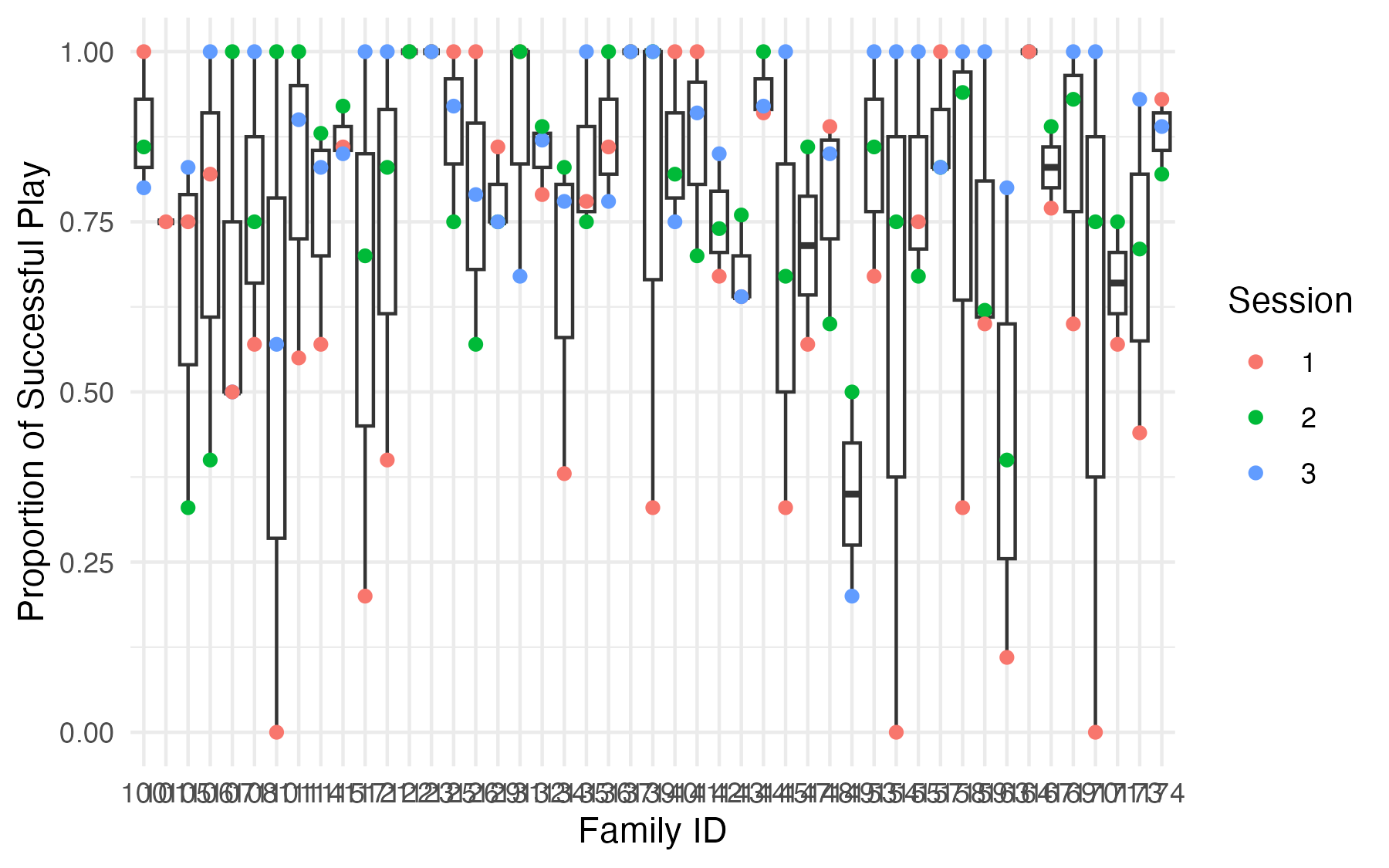


*Note.* Each boxplot represents the distribution of one family’s session-level engagement proportion.

**Video Chat Experience Models**

We pre-registered the inclusion of an interaction term for infant age and video chat frequency. The models with that interaction are presented below.

**Table S1**

*Final Models with the Video Chat Experience Interaction*

|  | Play frequency | | | | Play repertoire | | | | Play proportion | | | | Infant initiation | | | | Positive engagement | | | |
| --- | --- | --- | --- | --- | --- | --- | --- | --- | --- | --- | --- | --- | --- | --- | --- | --- | --- | --- | --- | --- |
|  | Est. | *SE* | *t* | *p* | Est. | *SE* | *t* | *p* | Est. | *SE* | *t* | *p* | Est. | *SE* | *t* | *p* | Est. | *SE* | *t* | *p* |
| (Intercept) | 0.37 | 5.01 | 0.07 | .941 | -1.09 | 1.56 | -0.70 | .485 | 0.19* | 0.08 | 2.31 | .023 | 0.24 | 0.24 | 0.99 | .323 | 0.42+ | 0.25 | 1.67 | .098 |
| Infant age (months) | 0.17 | 0.42 | 0.41 | .679 | 0.23+ | 0.13 | 1.77 | .080 | -0.01 | 0.01 | -1.17 | .243 | 0.01 | 0.02 | 0.29 | .773 | 0.03 | 0.02 | 1.64 | .103 |
| Video chat frequency | 0.61 | 1.92 | 0.32 | .752 | 0.97 | 0.61 | 1.60 | .111 | -0.03 | 0.03 | -0.91 | .366 | -0.12 | 0.09 | -1.36 | .176 | 0.04 | 0.09 | 0.47 | .642 |
| VC Experience (Age X Frequency) | 0.03 | 0.16 | 0.21 | .834 | -0.05 | 0.05 | -1.09 | .276 | 0.00 | 0.00 | 1.19 | .235 | 0.01 | 0.01 | 0.96 | .337 | 0.00 | 0.01 | -0.60 | .547 |
| Session duration | 0.19* | 0.08 | 2.38 | .019 | 0.07** | 0.03 | 2.76 | .007 |  |  |  |  | 0.00 | 0.00 | 0.02 | .983 | 0.00 | 0.01 | -0.27 | .789 |
| Play frequency |  |  |  |  |  |  |  |  |  |  |  |  | 0.00 | 0.01 | 0.11 | .910 | 0.01 | 0.00 | 1.30 | .195 |
| Play repertoire |  |  |  |  |  |  |  |  |  |  |  |  | -0.01 | 0.02 | -0.47 | .638 | 0.01 | 0.01 | 0.49 | .624 |
| Play proportion |  |  |  |  |  |  |  |  |  |  |  |  | -0.29 | 0.32 | -0.89 | .376 | -0.30 | 0.38 | -0.77 | .443 |
| SD (Familynum) | 2.37 |  |  |  | 1.07 |  |  |  | 0.05 |  |  |  | 0.10 |  |  |  |  |  |  |  |
| SD (Obs.) | 4.22 |  |  |  | 1.24 |  |  |  | 0.07 |  |  |  | 0.19 |  |  |  |  |  |  |  |
| Num.Obs. | 132 |  |  |  | 132 |  |  |  | 132 |  |  |  | 132 |  |  |  | 132 |  |  |  |
| R2 Marg. | 0.110 |  |  |  | 0.132 |  |  |  | 0.016 |  |  |  | 0.140 |  |  |  |  |  |  |  |
| R2 Cond. | 0.324 |  |  |  | 0.501 |  |  |  | 0.334 |  |  |  | 0.326 |  |  |  |  |  |  |  |
| AIC | 804.6 |  |  |  | 514.8 |  |  |  | -249.1 |  |  |  | 39.9 |  |  |  | 230.0 |  |  |  |
| ICC | 0.2 |  |  |  | 0.4 |  |  |  | 0.3 |  |  |  | 0.2 |  |  |  |  |  |  |  |
| RMSE | 3.86 |  |  |  | 1.08 |  |  |  | 0.06 |  |  |  | 0.18 |  |  |  |  |  |  |  |
